# Supplementary material for: N-glycosylation of the protein disulfide isomerase Pdi1 ensures full Ustilago maydis virulence
Source: PLoS Pathog. 2019 Nov 15;15(11):e1007687. doi: 10.1371/journal.ppat.1007687 (PMC6881057; doi:10.1371/journal.ppat.1007687)
Supplement: S1 Fig — (DOCX) [file ppat.1007687.s001.docx]

S1 Fig. Perl scripts used to identify O and N-glycoproteins in U. maydis genome are available in <https://github.com/ajperezpulido/glycosilation>
