# Supplementary figures and images for: N-glycosylation of the protein disulfide isomerase Pdi1 ensures full Ustilago maydis virulence
Source: PLoS Pathog. 2019 Nov 15;15(11):e1007687. doi: 10.1371/journal.ppat.1007687 (PMC6881057; doi:10.1371/journal.ppat.1007687)

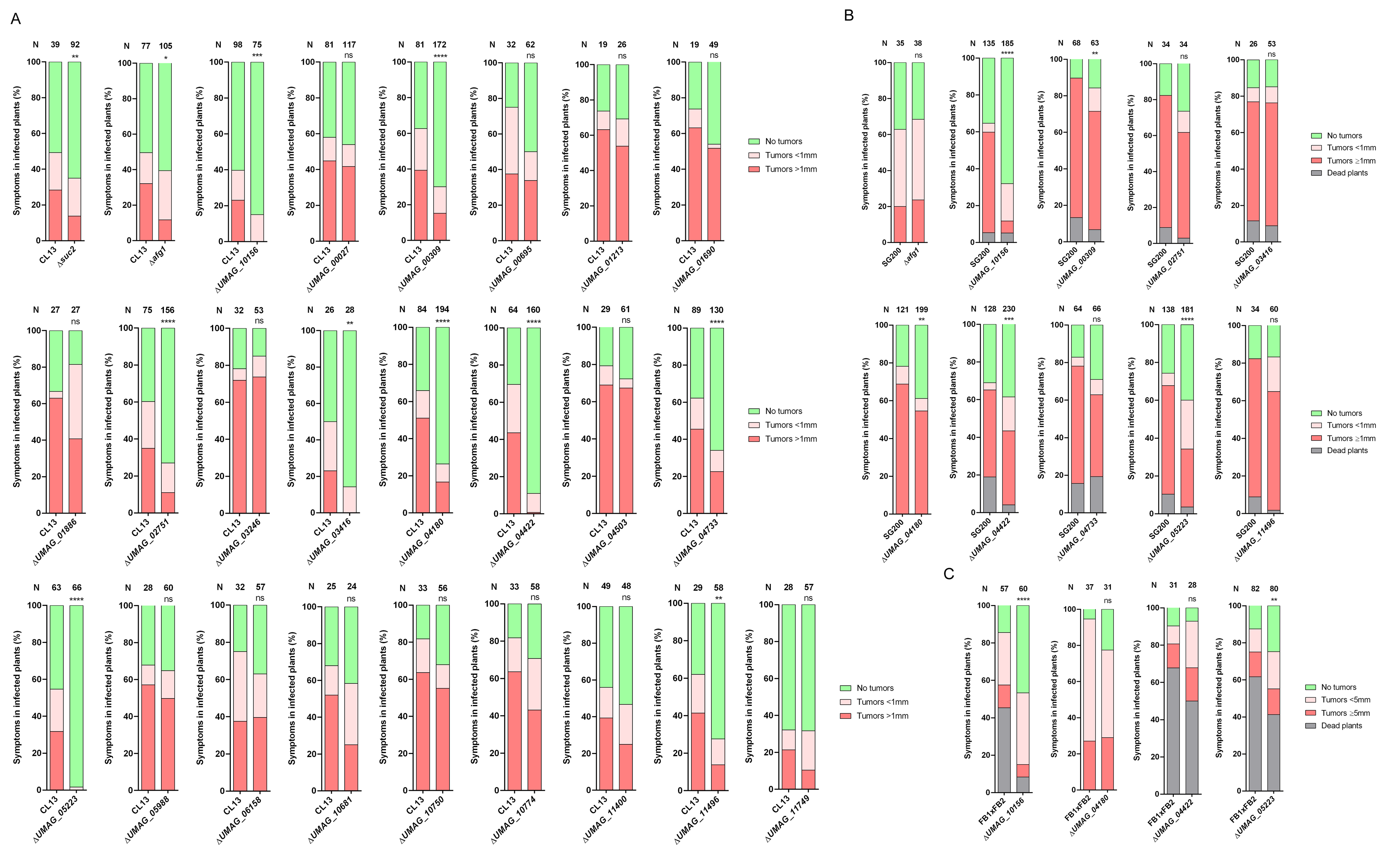

Supplement: S2 Fig — Deletions and infections were first carried out in the CL13 strain (A). Deletions showing a statistically significant reduction in virulence were then assayed in SG200 (B) and subsequently in FB1 and FB2 compatible strains (C). Total number of plants infected is indicated above each column. The Mann-Whitney statistical test was performed (ns: not statistically significant; * for p-value < 0.05; ** for p-value < 0.01; *** for p-value < 0.005; **** for p-value < 0.0001). (TIF) [file ppat.1007687.s002.tif]

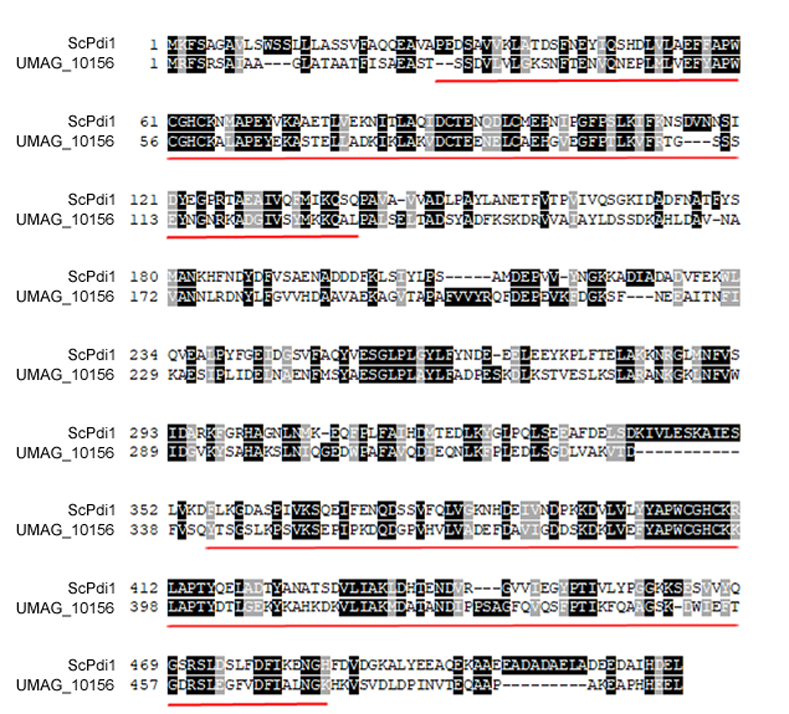

Supplement: S3 Fig — Alignment of Pdi1 sequence from S. cerevisiae and UMAG_10156 from U. maydis using T-Coffee Server and BoxShade Server. Thioredoxin domains are indicated by red lines. (TIF) [file ppat.1007687.s003.tif]

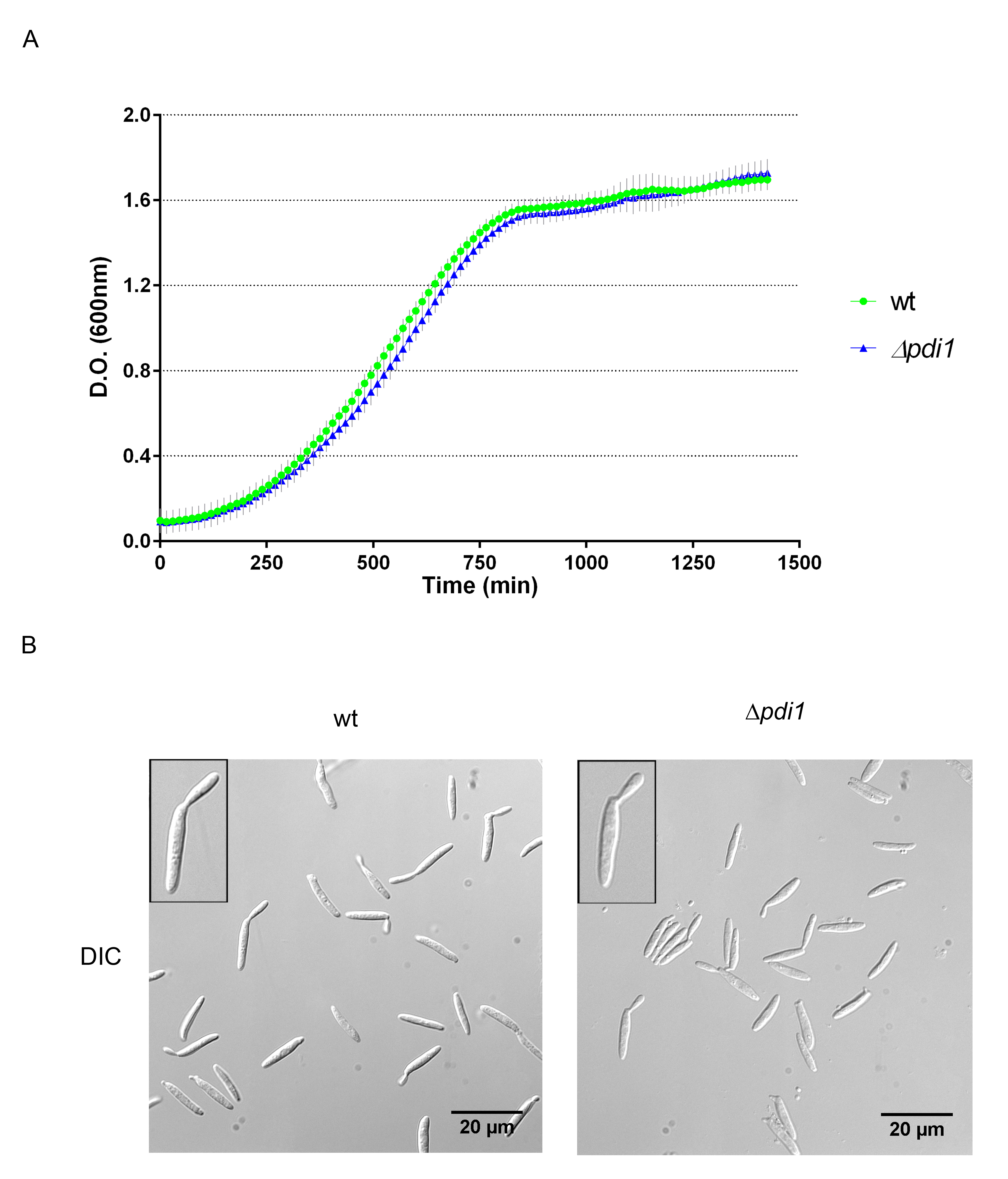

Supplement: S4 Fig — (A) Growth rate of SG200 and SG200 Δpdi1 in liquid rich media YEPSL. (B) SG200 and Δpdi1 cells observed by DIC microscopy do not show any defect in size and morphology. (TIF) [file ppat.1007687.s004.tif]

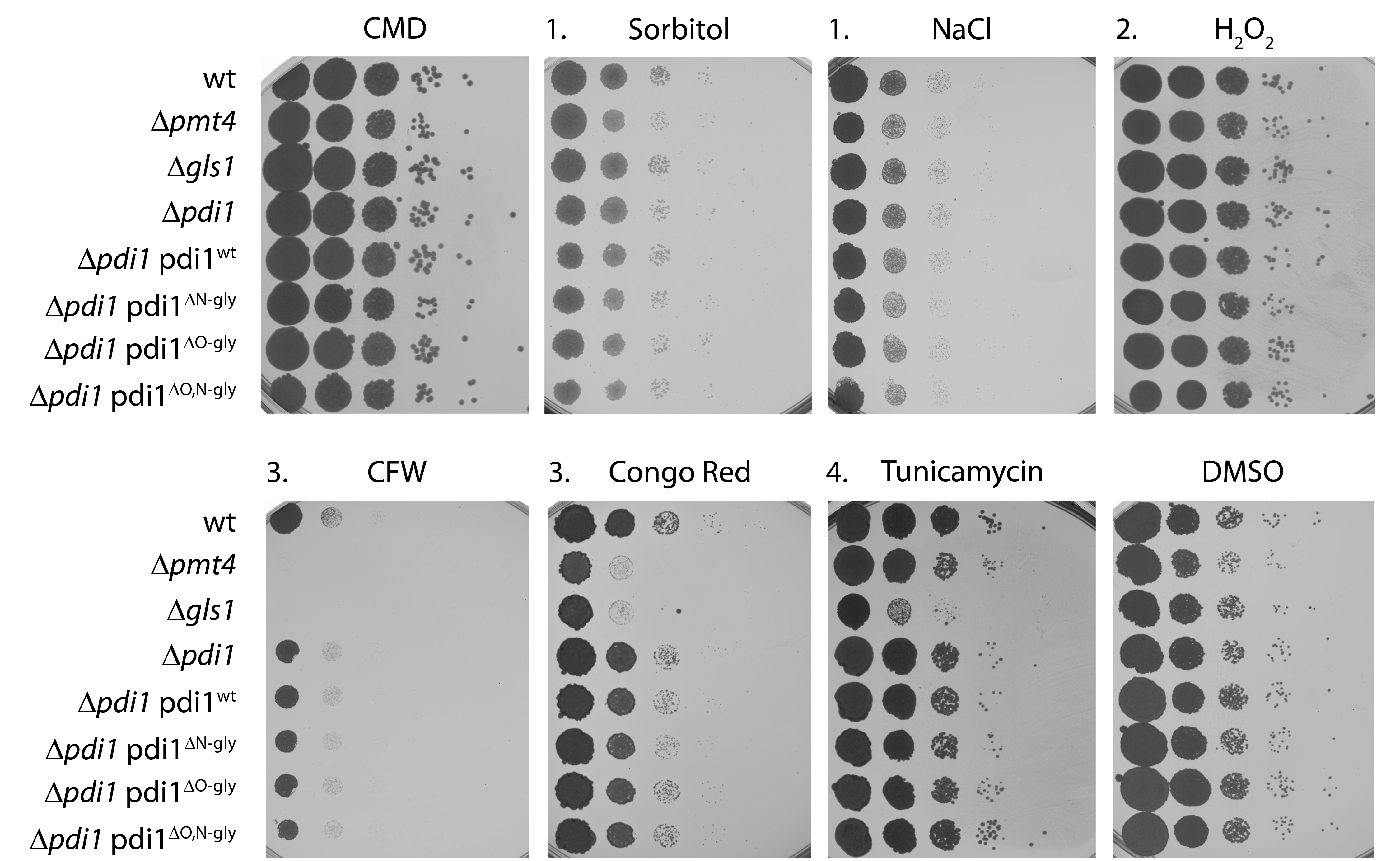

Supplement: S5 Fig — Osmotic (1) and oxidative (2) stress, cell wall integrity (3) and ER stress (4) assays were performed in CM plates supplemented with 2% D-glucose and Sorbitol 1M, NaCl 1M, H2O2 1.5 mM, calcofluor white (CFW) 40 μg/ml, Congo Red 50 μg/ml, Tunicamycin 1 μg/ml and 2% DMSO as Tunicamycin solvent control. (TIF) [file ppat.1007687.s005.tif]

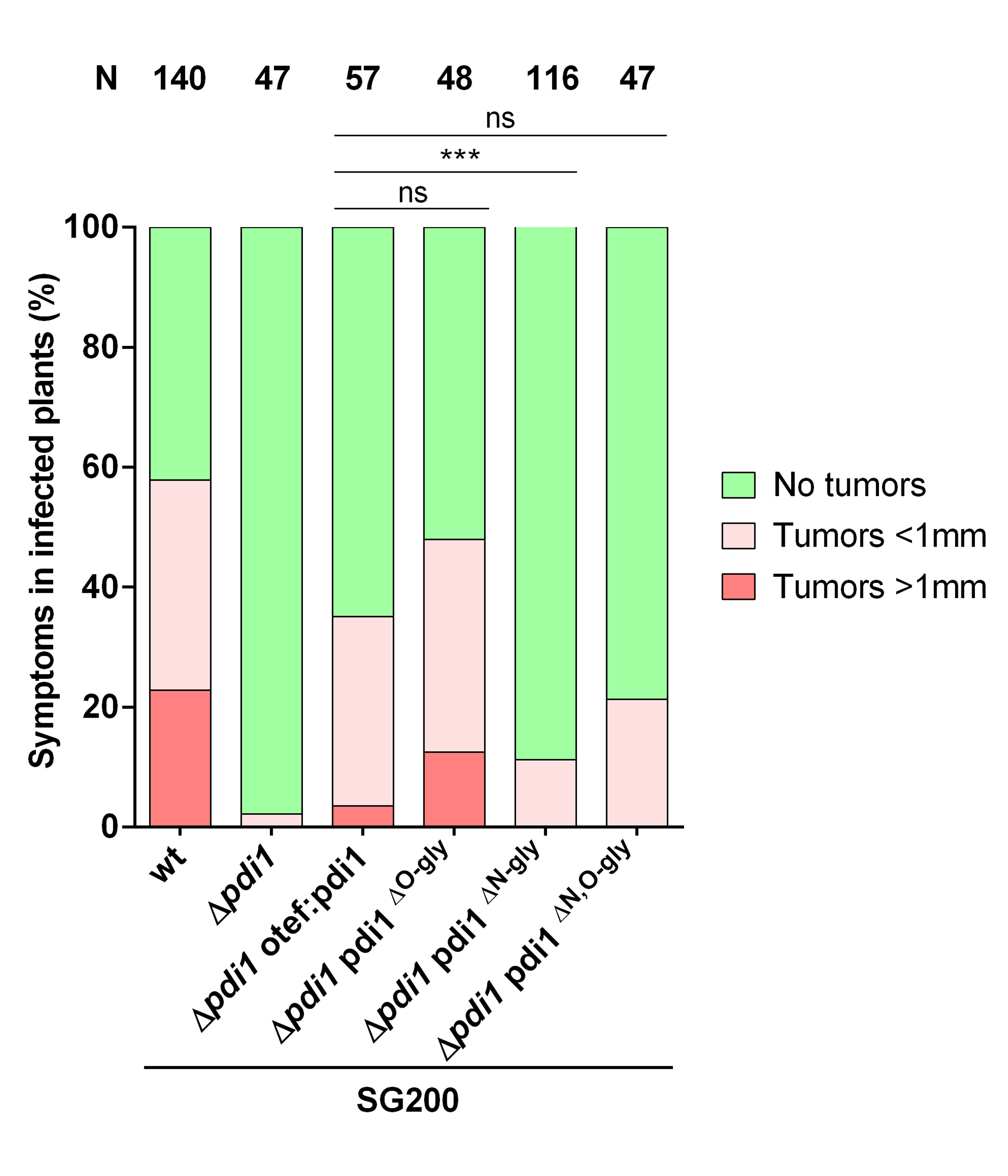

Supplement: S6 Fig — The percentage of symptoms in maize plants infected with the indicated strains at 14 dpi. The total number of infected plants is indicated above each column. Mann-Whitney statistical test was performed (ns: not statistically significant; *** for p-value < 0.005). (TIF) [file ppat.1007687.s006.tif]
