# Supplementary material for: N-glycosylation of the protein disulfide isomerase Pdi1 ensures full Ustilago maydis virulence
Source: PLoS Pathog. 2019 Nov 15;15(11):e1007687. doi: 10.1371/journal.ppat.1007687 (PMC6881057; doi:10.1371/journal.ppat.1007687)
Supplement: S2 Table — (DOCX) [file ppat.1007687.s008.docx]

**S2 Table. List of strains used in this study.**

| **Strain** | **Relevant Genotype** | **Reference** |
| --- | --- | --- |
| FB1 | a1 b1 | (Banuett and Herskowitz 1989) |
| FB2 | a2 b2 | (Banuett and Herskowitz 1989) |
| FB1 Δ*pmt4* | a1 b1 *Δpmt4* | (4) |
| FB1 *Δgls1* | a1 b1 *Δgls1* | (5) |
| FB1 *Δpdi1* | a1 b1 *Δpdi1* | This work |
| FB2 *Δpdi1* | a2 b2 Δpdi1 | This work |
| FB1 *ΔUMAG_04180* | a1 b1 *ΔUMAG_04180* | This work |
| FB2 *ΔUMAG_04180* | a2 b2 *ΔUMAG_04180* | This work |
| FB1 *ΔUMAG_04422* | a1 b1 *ΔUMAG_04422* | This work |
| FB2 *ΔUMAG_04422* | a2 b2 *ΔUMAG_04422* | This work |
| FB1 *ΔUMAG_05223* | a1 b1 *ΔUMAG_05223* | This work |
| FB2 *ΔUMAG_05223* | a2 b2 *ΔUMAG_05223* | This work |
| FB1 Biz1 | a1 b1 P_crg_:Biz1 | (Flor-Parra et al., 2006) |
| FB1 Biz1 *Δpmt4* | a1 b1 P_crg_:Biz1 *Δpmt4* | This work |
| FB1 Biz1 *Δgls1* | a1 b1 P_crg_:Biz1 *Δgls1* | This work |
| FB1 Biz1 *Δpdi1* | a1 b1 P_crg_:Biz1 *Δpdi1* | This work |
| SG200 | a1 mfa2 bW2 bE1 | (Bolker et al., 1995) |
| SG200 *Δpmt4* | a1 mfa2 bW2 bE1 *Δpmt4* | (4) |
| SG200 *Δgls1* | a1 mfa2 bW2 bE1 *Δgls1* | (5) |
| SG200 *Δafg1* | a1 mfa2 bW2 bE1 *Δafg1* | This work |
| SG200 *Δpdi1* | a1 mfa2 bW2 bE1 *Δpdi1* | This work |
| SG200 *ΔUMAG_00309* | a1 mfa2 bW2 bE1 *ΔUMAG_00309* | This work |
| SG200 *ΔUMAG_01209* | a1 mfa2 bW2 bE1 *ΔUMAG_01209* | This work |
| SG200 *ΔUMAG_02751* | a1 mfa2 bW2 bE1 *ΔUMAG_02751* | This work |
| SG200 *ΔUMAG_03416* | a1 mfa2 bW2 bE1 *ΔUMAG_03416* | This work |
| SG200 *ΔUMAG_04180* | a1 mfa2 bW2 bE1 *ΔUMAG_04180* | This work |
| SG200 *ΔUMAG_04270* | a1 mfa2 bW2 bE1 *ΔUMAG_04270* | This work |
| SG200 *ΔUMAG_04382* | a1 mfa2 bW2 bE1 *ΔUMAG_04382* | This work |
| SG200 *ΔUMAG_04422* | a1 mfa2 bW2 bE1 *ΔUMAG_04422* | This work |
| SG200 *ΔUMAG_04733* | a1 mfa2 bW2 bE1 *ΔUMAG_04733* | This work |
| SG200 *ΔUMAG_05223* | a1 mfa2 bW2 bE1 *ΔUMAG_05223* | This work |
| SG200 *ΔUMAG_11496* | a1 mfa2 bW2 bE1 *ΔUMAG_11496* | This work |
| SG200 3xGFP | a1 mfa2 bW2 bE1 P_otef_:3xGFP | This work |
| SG200 2xRFP *Δpdi1* | a1 mfa2 bW2 bE1 P_otef_:2xRFP *Δpdi1* | This work |
| SG200 *Δpdi1* pOtef:pdi1^wt^ | a1 mfa2 bW2 bE1 *Δpdi1* P_otef_:pdi1 | This work |
| SG200 *Δpdi1* pOtef:pdi1 ^ΔO-gly^ | a1 mfa2 bW2 bE1 *Δpdi1* P_otef_:pdi1 ^ΔO-gly^ | This work |
| SG200 *Δpdi1* pOtef:pdi1 ^ΔN-gly^ | a1 mfa2 bW2 bE1 *Δpdi1* P_otef_:pdi1 ^ΔN-gly^ | This work |
| SG200 *Δpdi1* pOtef:pdi1 ^ΔN,O-gly^ | a1 mfa2 bW2 bE1 *Δpdi1* P_otef_:pdi1 ^ΔN,O-gly^ | This work |
| SG200 *Δpdi1* pPdi1:pdi1^wt^ | a1 mfa2 bW2 bE1 *Δpdi1* P_pdi1_:pdi1 | This work |
| SG200 *Δpdi1* pPdi1:pdi1 ^ΔO-gly^ | a1 mfa2 bW2 bE1 *Δpdi1* P_pdi1_:pdi1 ^ΔO-gly^ | This work |
| SG200 *Δpdi1* pPdi1:pdi1 ^ΔN-gly^ | a1 mfa2 bW2 bE1 *Δpdi1* P_pdi1_:pdi1 ^ΔN-gly^ | This work |
| SG200 *Δpdi1* pPdi1:pdi1 ^ΔN,O-gly^ | a1 mfa2 bW2 bE1 *Δpdi1* P_pdi1_:pdi1 ^ΔN,O-gly^ | This work |
| SG200 pdi1:GFP | a1 mfa2 bW2 bE1 P_otef_:pdi1-GFP | This work |
| SG200 *Δpdi1* pdi1 ^ΔN-gly^:GFP | a1 mfa2 bW2 bE1 *Δpdi1* P_otef_:pdi1 ^ΔN-gly^-GFP | This work |
| SG200 mrfp:HDEL | a1 mfa2 bW2 bE1 P_otef_:cal^s^:mrfp-HDEL | (Theisen et al., 2008) |
| CL13 | a1 bE1 bW2 | (Bolker et al., 1995) |
| CL13 *Δsuc2* | a1 bE1 bW2 *Δsuc2* | This work |
| CL13 *Δafg1* | a1 bE1 bW2 *Δafg1* | This work |
| CL13 *Δpdi1* | a1 bE1 bW2 *Δpdi1* | This work |
| CL13 *ΔUMAG_00027* | a1 bE1 bW2 *ΔUMAG_00027* | This work |
| CL13 *ΔUMAG_00309* | a1 bE1 bW2 *ΔUMAG_00309* | This work |
| CL13 *ΔUMAG_00695* | a1 bE1 bW2 *ΔUMAG_00695* | This work |
| CL13 *ΔUMAG_01213* | a1 bE1 bW2 *ΔUMAG_01213* | This work |
| CL13 *ΔUMAG_01690* | a1 bE1 bW2 *ΔUMAG_01690* | This work |
| CL13 *ΔUMAG_01886* | a1 bE1 bW2 *ΔUMAG_01886* | This work |
| CL13 *ΔUMAG_02751* | a1 bE1 bW2 *ΔUMAG_02751* | This work |
| CL13 *ΔUMAG_03246* | a1 bE1 bW2 *ΔUMAG_03246* | This work |
| CL13 *ΔUMAG_03416* | a1 bE1 bW2 *ΔUMAG_03416* | This work |
| CL13 *ΔUMAG_04180* | a1 bE1 bW2 *ΔUMAG_04180* | This work |
| CL13 *ΔUMAG_04422* | a1 bE1 bW2 *ΔUMAG_04422* | This work |
| CL13 *ΔUMAG_04503* | a1 bE1 bW2 *ΔUMAG_04503* | This work |
| CL13 *ΔUMAG_04733* | a1 bE1 bW2 *ΔUMAG_04733* | This work |
| CL13 *ΔUMAG_05223* | a1 bE1 bW2 *ΔUMAG_05223* | This work |
| CL13 *ΔUMAG_05988* | a1 bE1 bW2 *ΔUMAG_05988* | This work |
| CL13 *ΔUMAG_06158* | a1 bE1 bW2 *ΔUMAG_06158* | This work |
| CL13 *ΔUMAG_10681* | a1 bE1 bW2 *ΔUMAG_10681* | This work |
| CL13 *ΔUMAG_10750* | a1 bE1 bW2 *ΔUMAG_10750* | This work |
| CL13 *ΔUMAG_10774* | a1 bE1 bW2 *ΔUMAG_10774* | This work |
| CL13 *ΔUMAG_11400* | a1 bE1 bW2 *ΔUMAG_11400* | This work |
| CL13 *ΔUMAG_11496* | a1 bE1 bW2 *ΔUMAG_11496* | This work |
| CL13 *ΔUMAG_11749* | a1 bE1 bW2 *ΔUMAG_11749* | This work |
