# Supplementary material for: N-glycosylation of the protein disulfide isomerase Pdi1 ensures full Ustilago maydis virulence
Source: PLoS Pathog. 2019 Nov 15;15(11):e1007687. doi: 10.1371/journal.ppat.1007687 (PMC6881057; doi:10.1371/journal.ppat.1007687)
Supplement: S3 Table — (DOCX) [file ppat.1007687.s009.docx]

**S3 Table. Primers used in this study.**

| **Primer name** | **Sequence (5' -> 3')** |
| --- | --- |
| suc2KO5-1 | CTGTAACGCTGAGTTGTTCG |
| suc2KO5-2 | CACGGCCTGAGTGGCCGGATCTGTGACAAGCAAGACG |
| suc2KO3-1 | GTGGCCATCTAGGCCTGACTTCATGCTCCGTCTCG |
| suc2KO3-2 | TGCGATGAACGAATGCTTGG |
| afg1KO5-1 | GATCGTTGAGAACCTGTGTCG |
| afg1KO5-2 | CACGGCCTGAGTGGCCTGTTGCAGAGGGTAGATACAGC |
| afg1KO3-1 | GTGGCCATCTAGGCCCATTGCATCGACCCTCAACAGC |
| afg1KO3-2 | GCCTTACTCCGCAACTTCAGC |
| pdi1KO5-1 | CCAATCGGAAGCAAGTCTCG |
| pdi1KO5-2 | CACGGCCTGAGTGGCCAGACAAGGTGGAAGAGCAAGG |
| pdi1KO3-1 | GTGGCCATCTAGGCCGATGTCGTCCCGATACATGC |
| pdi1KO3-2 | AAGGCTGGGATCTTTCCGTAGG |
| UMAG_00027KO5-1 | AGACCAAAGGAGAGAAGCAGACC |
| UMAG_00027KO5-2 | CACGGCCTGAGTGGCCTTGGTTAGTGGGCGGAGATGG |
| UMAG_00027KO3-1 | GTGGCCATCTAGGCCACATCTTGGGCCTGATAGACG |
| UMAG_00027KO3-2 | TATTTCGTTCCGTTGCGTTCTGG |
| UMAG_00309KO5-1 | ACGACTGGCTGCTAAACAGG |
| UMAG_00309KO5-2 | CACGGCCTGAGTGGCCTGTTGAGAGAGATGCTTTCCTGC |
| UMAG_00309KO3-1 | GTGGCCATCTAGGCCACTGCGTCGCATGTATTTCC |
| UMAG_00309KO3-2 | TTCGTGAACCCATCTCCAGC |
| UMAG_00695KO5-1 | AAGTACATGGCGGCAGAAGC |
| UMAG_00695KO5-2 | CACGGCCTGAGTGGCCTGTGAAGCAGGTATGAAGGTGG |
| UMAG_00695KO3-1 | GTGGCCATCTAGGCCCTTACATCCTCAGCCTTGAGC |
| UMAG_00695KO3-2 | TCTGGTCTCGGCTTCTAGTCG |
| UMAG_01209KO5-1 | GGTAGAATTATGGTAGTTGGGTGG |
| UMAG_01209KO5-2 | CACGGCCTGAGTGGCCAATTGAAGGAGATGGAACGAGACG |
| UMAG_01209KO3-1 | GTGGCCATCTAGGCCTTCTTCCACCTCTCTTTAGGTCTCG |
| UMAG_01209KO3-2 | TGTACCGGGAGAACATGAAGG |
| UMAG_01213KO5-1 | GAGCGGAACTAGATGCAACAACG |
| UMAG_01213KO5-2 | CACGGCCTGAGTGGCCAGACACATAGATGTAGGCACGATGG |
| UMAG_01213KO3-1 | GTGGCCATCTAGGCCGAGTCCAGTAAAGGAAGTGGGAGG |
| UMAG_01213KO3-2 | TCTGAGACCATCCATCTGTCC |
| UMAG_01690KO5-1 | AAGTACATGGCGGCAGAAGC |
| UMAG_01690KO5-2 | CACGGCCTGAGTGGCCTGTGAAGCAGGTATGAAGGTGG |
| UMAG_01690KO3-1 | GTGGCCATCTAGGCCCTTACATCCTCAGCCTTGAGC |
| UMAG_01690KO3-2 | TCTGGTCTCGGCTTCTAGTCG |
| UMAG_01886KO5-1 | TGTTTGCAGACGGCTTCTCG |
| UMAG_01886KO5-2 | CACGGCCTGAGTGGCCCTGAAAGCGAAGCGAGCTGC |
| UMAG_01886KO3-1 | GTGGCCATCTAGGCCGATCTAAACAGGACACACTGGTCG |
| UMAG_01886KO3-2 | GCTGATTCCAGCTTCGGACG |
| UMAG_02751KO5-1 | TTGGTGAAGCGCGAGATAGC |
| UMAG_02751KO5-2 | CACGGCCTGAGTGGCCGCTCACCGTGCGTTGTTTATACC |
| UMAG_02751KO3-1 | GTGGCCATCTAGGCCGCTTGATAAGGCGATAGTAGTGC |
| UMAG_02751KO3-2 | GATTCGCAAGAGGAAACCGAGG |
| UMAG_03246KO5-1 | GTGAACGAGTAAGCACGGAAGC |
| UMAG_03246KO5-2 | CACGGCCTGAGTGGCCATTTGGGCAGATCCTGAGAGTGG |
| UMAG_03246KO3-1 | GTGGCCATCTAGGCCAGGAAATTGTTTGACCTGGTCG |
| UMAG_03246KO3-2 | CGCTAAATTCAGACGCGAGATGG |
| UMAG_03416KO5-1 | GTAGCGGTTGTGGAACCAGG |
| UMAG_03416KO5-2 | CACGGCCTGAGTGGCCCACCAGTTCTCACGTCTCGACC |
| UMAG_03416KO3-1 | GTGGCCATCTAGGCCTTTGGCCTCATCGAGTTTCACC |
| UMAG_03416KO3-2 | CTGGTAAGATGTTTGCTCTGCTCG |
| UMAG_04180KO5-1 | CCACATGGTTTCTTCCAACGAGC |
| UMAG_04180KO5-2 | CACGGCCTGAGTGGCCCACTATCTTGTGCCTATACGTCC |
| UMAG_04180KO3-1 | GTGGCCATCTAGGCCTATACCATTCGTCCTTCTTCCTGC |
| UMAG_04180KO3-2 | CGACTTCCTCTGGTTCATTCACC |
| UMAG_04270KO5-1 | ACCAAACACCACTTTCGACATCC |
| UMAG_04270KO5-2 | CACGGCCTGAGTGGCCTGATGGTGATGTGAAGTGATGACG |
| UMAG_04270KO3-1 | GTGGCCATCTAGGCCCCATTCAGGCACTCTTCACG |
| UMAG_04270KO3-2 | CTGGACGACCCAAAGTGACC |
| UMAG_04382KO5-1 | ATGACAGAAGTCACAGGCTCACC |
| UMAG_04382KO5-2 | CACGGCCTGAGTGGCCGCATCGCACGCTAAATACCACC |
| UMAG_04382KO3-1 | GTGGCCATCTAGGCCGTGCCACGCTACCAATCACG |
| UMAG_04382KO3-2 | CTGTACTACTCGATAGCCGCTTGG |
| UMAG_04422KO5-1 | TATCACAGACGCCACGCTCG |
| UMAG_04422KO5-2 | CACGGCCTGAGTGGCCGATCACAACACAAGAGAGGAGGAGG |
| UMAG_04422KO3-1 | GTGGCCATCTAGGCCTTCGTGATTGAAGATGCGTG |
| UMAG_04422KO3-2 | CAAGTGTGAGTTGGGAGTGACG |
| UMAG_04503KO5-1 | AACGTACAACGTGCAACCAGG |
| UMAG_04503KO5-2 | CACGGCCTGAGTGGCCGAAGATGGAATCGCCTAGTGC |
| UMAG_04503KO3-1 | GTGGCCATCTAGGCCACTCATCGTTGCTCACTGTGG |
| UMAG_04503KO3-2 | CGAAGGTATCCCGACTGAAGC |
| UMAG_04733KO5-1 | AACACGCTGTTCCTGATCTGG |
| UMAG_04733KO5-2 | CACGGCCTGAGTGGCCGAGATTGTCTAGTGTCTTGCCTGG |
| UMAG_04733KO3-1 | GTGGCCATCTAGGCCAACAGGGCTCTTCTCTTTGG |
| UMAG_04733KO3-2 | GAGGCTGCGTATGATCTCACC |
| UMAG_05223KO5-1 | CGATGAAAGGGTGCAGATGG |
| UMAG_05223KO5-2 | CACGGCCTGAGTGGCCACCTGTCACTTTCTTGTCCTTCC |
| UMAG_05223KO3-1 | GTGGCCATCTAGGCCCTCAAGAGAAGAGGTCAAGACG |
| UMAG_05223KO3-2 | CTCAACCGTCAAGTTCTTGC |
| UMAG_05988KO5-1 | ATTCGACGACTGTGTGATGG |
| UMAG_05988KO5-2 | CACGGCCTGAGTGGCCTGTGACCCGCTTTGTTTGTTCC |
| UMAG_05988KO3-1 | GTGGCCATCTAGGCCCGTGTGTAGAGAAGAACGTCG |
| UMAG_05988KO3-2 | GTCGTTCTCGGATGGCATCG |
| UMAG_06158KO5-1 | GGCAATCACATCACAGAATCACG |
| UMAG_06158KO5-2 | CACGGCCTGAGTGGCCGAAGGATGCAGAAGTGGATGG |
| UMAG_06158KO3-1 | GTGGCCATCTAGGCCGCATTCACGATTGACTAGATCC |
| UMAG_06158KO3-2 | CTGAAATACAGAAGAGACAGCTCG |
| UMAG_10681KO5-1 | AGACAAGTCACGAGAACGATGG |
| UMAG_10681KO5-2 | CACGGCCTGAGTGGCCGGTTGGTGTGTCTGGATGACG |
| UMAG_10681KO3-1 | GTGGCCATCTAGGCCGAAGCTCGGTTTGGGTTGATCC |
| UMAG_10681KO3-2 | GGATCGTCTAGCTTGTCTGG |
| UMAG_10750KO5-1 | CTACCTTGTACGCCTGCTGC |
| UMAG_1075KO5-2 | CACGGCCTGAGTGGCCGAATGTCAAACTGTGCGCTCACC |
| UMAG_10750KO3-1 | GTGGCCATCTAGGCCCCAAGCTGCCTCAGTAGACC |
| UMAG_10750KO3-2 | TGTAGCAGCTCGCGTATCTGG |
| UMAG_10774KO5-1 | CGAGCCGTGTCTTCATCTAGC |
| UMAG_10774KO5-2 | CACGGCCTGAGTGGCCGAGACTGAGCGATTTCGACG |
| UMAG_10774KO3-1 | GTGGCCATCTAGGCCGAATGTCATTGAACCCGAAGTGC |
| UMAG_10774KO3-2 | ACACCGGATTTCTAGTCATGG |
| UMAG_11400KO5-1 | ACTCGTGACTCTAACTCTTGG |
| UMAG_11400KO5-2 | CACGGCCTGAGTGGCCCAAGAGGAGAGGATGAGCTAGG |
| UMAG_11400KO3-1 | GTGGCCATCTAGGCCGTGGTAAGTGCAGTGTGATGG |
| UMAG_11400KO3-2 | CCGATCTGAGCCCTACTAGC |
| UMAG_11496KO5-1 | AAAGCCCAGCTCTCTGGAGG |
| UMAG_11496KO5-2 | CACGGCCTGAGTGGCCTGTGGAGAAGAAAGCACCAAGG |
| UMAG_11496KO3-1 | GTGGCCATCTAGGCCCAACGCTCATTCGAGTCTCACC |
| UMAG_11496KO3-2 | CGAGAAGTCCAACAGCACAGG |
| UMAG_11749KO5-1 | GCTTGCTTTCGTGTTAAGCTGC |
| UMAG_11749KO5-2 | CACGGCCTGAGTGGCCGAATGGCCGTCAAATGATGTGG |
| UMAG_11749KO3-1 | GTGGCCATCTAGGCCCAACACACTGCTCAGATCATCC |
| UMAG_11749KO3-2 | CCACTGCATTTGTTGGAGAACG |
| pdi1StartXmaI | AATCCCGGGCTGCAGGAATTCGATCCCATGCGATTCTCTCGTTCCGCCATCGCGG |
| pdi1StopNotI | TAAGCGGCCGCTCATAGCTCCTCGTGGTGAGGAGCCTCC |
| pdi1StopNcoI | TCACCATGGCTCTTAGCTCCTCGTGGTGAGG |
| PDI1Asn36mut-1 | TCGGCAAATCCCAATTCACCGAGAATGTTCAGAACG |
| PDI1Asn36mut-2 | TTCTCGGTGAATTGGGATTTGCCGAGC |
| PDI1Asn484mut-1 | TCGATCCGATCCAAGTTACCGAGCAGG |
| PDI1Asn484mut-2 | TGCTCGGTAACTTGGATCGGATCGAGGTCGACC |
| PDI1Asn484mutThr486mut-1 | TCGATCCGATCCAAGTTGCCGAGCAGG |
| PDI1Asn484mutThr486mut-2 | TGCTCGGCAACTTGGATCGGATCGAGGTCGACC |
| pdi1ORF5 | ATAGGCCTGAGTGGCCATGCGATTCTCTCGTTCCGCCATCGC |
| pdi1ORF3 | ATAGGCCGCGTTGGCCCCTAGCTCCTCGTGGTGAGGAGCCTCC |
| RT-PPI1-5 | CGAGAACGAGGGCACCAA |
| RT-PPI1-3 | GCGAAAAAGCGTTTAAAGAACAC |
| pdi1 RT-fwd | TGGTTTCCAGGTGCAGTCATT |
| pdi1 RT-rev | TGAACTCGATCCAGTCCTTGC |
| Gapdh-F | CTTCGGCATTGTTGAGGGTTTG |
| Gapdh-R | TCCTTGGCTGAGGGTCCGTC |
| PDI1SeqI | GCGAGACGTTCCAATAAAGG |
| PDI1SeqII | GTCCAAGGACCGTGTTGTCG |
| pPdi1-F-NEB | ACACTATAGAACTCGAGCAGTTGCTGCGTATGTGTGAC |
| pPdi1-R-NEB | GGATCGAATTCCTGCAGCCCGTTGAAGAGTAGGCTCTAC |
