# Supplementary material for: N-glycosylation of the protein disulfide isomerase Pdi1 ensures full Ustilago maydis virulence
Source: PLoS Pathog. 2019 Nov 15;15(11):e1007687. doi: 10.1371/journal.ppat.1007687 (PMC6881057; doi:10.1371/journal.ppat.1007687)
Supplement: S4 Table — The Mann-Whitney statistical test was applied to each strain versus Δpdi1 and Δpdi1 pPdi1:pdi1 (pdi1wt) for each independent experiment (R1, R2 and R3). P-values obtained are listed in this table. Statistical significance is indicated by p-values in bold. (DOCX) [file ppat.1007687.s010.docx]

**S4 Table. Mann-Whitney statistical test individually performed for each pathogenic assay in Fig 7B.** The Mann-Whitney statistical test was applied to each strain versus Δ*pdi1* and Δ*pdi1* pPdi1:pdi1 (pdi1^wt^) for each independent experiment (R1, R2 and R3). P-values obtained are listed in this table.

Statistical significance is indicated by p-values in bold.

| R1 |  | wt | Δ*pdi1* | pdi1^wt^ | pdi1^∆O-gly^ | pdi1^∆N-gly^ | pdi1^∆N,O-gly^ |
| --- | --- | --- | --- | --- | --- | --- | --- |
|  | Δ*pdi1* | **< 0,0001** | n/a | **< 0,0001** | **0,0008** | 0,0663 | **0,0192** |
|  | pdi1^wt^ | 0,9993 | **< 0,0001** | n/a | 0,3488 | **0,0237** | 0,0584 |
|  |  |  |  |  |  |  |  |
| R2 |  | wt | Δ*pdi1* | pdi1^wt^ | pdi1^∆O-gly^ | pdi1^∆N-gly^ | pdi1^∆N,O-gly^ |
|  | Δ*pdi1* | **< 0,0001** | n/a | **0,0007** | **0,0002** | 0,574 | **0,0007** |
|  | pdi1^wt^ | 0,2674 | **0,0007** | n/a | 0,904 | **0,0239** | 0,6635 |
|  |  |  |  |  |  |  |  |
| R3 |  | wt | Δ*pdi1* | pdi1^wt^ | pdi1^∆O-gly^ | pdi1^∆N-gly^ | pdi1^∆N,O-gly^ |
|  | Δ*pdi1* | **< 0,0001** | n/a | **0,0009** | **0,0037** | 0,3954 | **0,0223** |
|  | pdi1^wt^ | 0,5496 | **0,0009** | n/a | 0,9032 | **0,0218** | 0,2788 |
